# The role of extrachromosomal DNA in tumorigenesis and progression

**DOI:** 10.3389/fonc.2025.1665024

**Published:** 2025-09-15

**Authors:** Xiaoyang Ma, Xiaolin Yu, Chuan Wu, Lixing Song

**Affiliations:** ^1^ Department of Clinical Laboratory, Zigong Fourth People’s Hospital, Zigong, China; ^2^ Sichuan Vocational College of Health and Rehabilitation, Zigong, China

**Keywords:** ecDNA, tumorigenesis, oncogene, heterogeneity, cancer progression

## Abstract

In tumors, extrachromosomal DNA (ecDNA) is an important driver of oncogene expression, genomic instability, the evolution of drug resistance, and poor patient prognosis. ecDNA is present in various tumors but is rarely found in normal cells. Here, we provide a detailed review of the structure, genetics, occurrence, outcomes, and functions of ecDNA, offering further reference for research on ecDNA.

## 1 Introduction

In 1965, Cox et al. (1) discovered a large number of small double chromatin bodies outside the chromosomes in five cases of pediatric embryonal tumors and one case of a rare type of adult bronchial carcinoma. In 1967, Radloff et al. (2) isolated and detected circular DNA of varying lengths in the HeLa cervical cancer cell line. The double chromatin bodies reported by Cox et al. (1) and the circular DNA reported by Radloff et al. (2) are both located extrachromosomally and are now collectively referred to as ecDNA. In 2024, Bailey et al. (3) analyzed whole-genome sequencing data from 14,778 tumor samples of 39 types and found that 17.1% of tumor samples contained ecDNA. The high detection rate of ecDNA in tumors has prompted extensive research to elucidate the role of ecDNA in tumorigenesis and progression. This article will comprehensively discuss the formation mechanisms, biological structure, genetic patterns, and functions of ecDNA in tumors, as well as its potential clinical applications, providing guidance for further research on ecDNA.

## 2 Structural and inheritance dynamics of ecDNA

### 2.1 Physical and functional architecture

**Figure 1:**
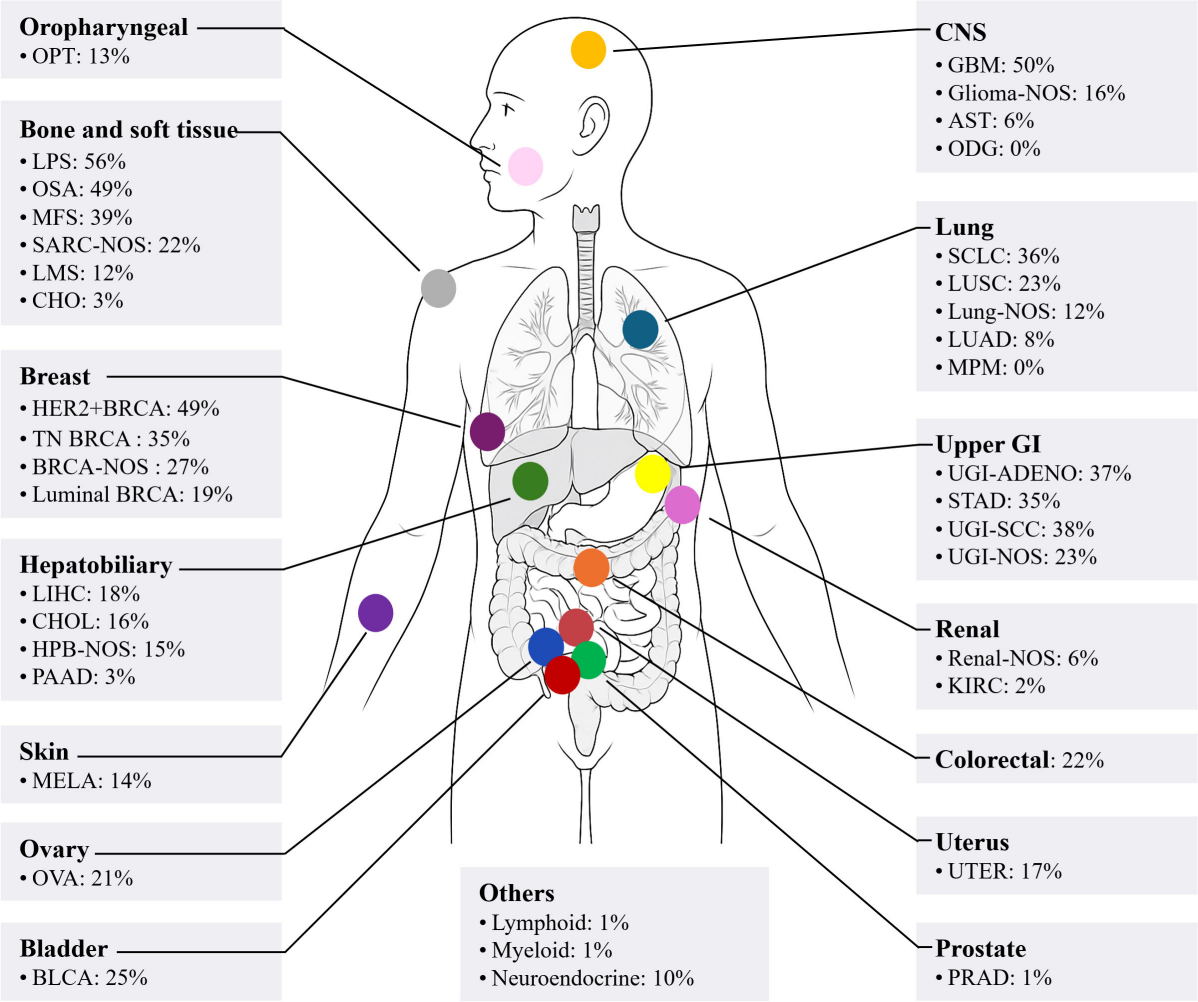
A body map of the prevalence of ecDNA in various cancers. OPT, oropharyngeal tumour; LPS, liposarcoma; OSA, primary conventional osteosarcoma; MFS, myxofibrosarcoma; SARC, sarcoma; NOS, not-otherwise specified; LMS, leiomyosarcoma; CHO, chordoma; BRCA, breast cancer; TN, triple negative; LIHC, liver hepatocellular carcinoma; CHOL, cholangiocarcinoma; HPB, hepatopancreatobiliary cancer; PAAD, pancreatic adenocarcinoma; MELA, malignant melanoma; OVA, ovarian cancer; BLCA, bladder cancer; CNS, central nervous system; GBM, glioblastoma; AST, astrocytoma; ODG, oligodendroglioma; SCLC, small cell lung cancer; LUSC, lung squamous cell carcinoma; LUAD, lung adenocarcinoma; MPM, malignant pleural mesothelioma; GI, gastrointestinal; UGI, upper gastrointestinal; ADENO, adenocarcinoma; STAD, stomach adenocarcinoma; SCC, squamous cell carcinoma; KIRC, clear cell renal cell carcinoma; UTER, endometrial cancer; PRAD, prostate adenocarcinoma. The data were extracted using Getdata Graph Digitizer ( *https://getdata-graph-digitizer.com/* ) from Bailey et al. (3), 2024, on June 6, 2025.

Approximately 30% of ecDNA exist in pairs within the nucleus, and thus, for a long time, they were referred to as double minutes (4). ecDNA has a complex structure, lacks centromeres, and can originate from multiple chromosomes (5). The frequency, copy number, and size of ecDNA vary greatly among different tumors (3). The prevalence of ecDNA across various cancers is shown in **Figure 1**. Typically, ecDNA exists in a circular form (4). However, ecDNA is distinct from extrachromosomal circular DNA (eccDNA). eccDNA has a small molecular weight (<1 kb), does not undergo amplification, does not contain complete gene sequences or regulatory elements, does not carry mutated genes, and can appear in normal tissues (5–11). ecDNA has a large molecular weight (>100 kb), undergoes clonal selection, possesses self-replication and amplification capabilities, can contain oncogenes, regulatory elements, recombinant genes, and mutated genes, and is rare in normal tissues (5, 6, 9–12).

ecDNA can carry a variety of common oncogenes, such as *MYC*, *MYCN*, *Jun*, *KRAS*, *MYCL*, *MDM2*, epidermal growth factor receptor (*EGFR*), fibroblast growth factor receptor 2 (*FGFR2*), platelet derived growth factor receptor alpha (*PDGFRA*), erb-b2 receptor tyrosine kinase 2 (*ERBB2*), and cyclin-dependent kinase 4 (*CDK4*), among others (13, 14). Even when carrying the same oncogene, ecDNA can exhibit substantial variability in both size and sequence composition (15). ecDNA with distinct sequence architectures are referred to as ecDNA species. ecDNA can simultaneously carry multiple oncogenes (13), and share adjacent regulatory regions (16). In addition, there exists a class of ecDNA that does not carry oncogenes but only carries promoters, enhancers, or long noncoding RNA (lncRNA) regulatory elements, referred to as regulatory ecDNA (3). Compared to ecDNA carrying oncogenes, regulatory ecDNA have a simpler structure, smaller size, and lower copy number (3). Whole-genome sequencing of human papillomavirus mediated oropharyngeal cancer (HPVOPC) revealed that HPVOPC contains ecDNA composed of host genome and HPV-host genome hybrids, and both types of ecDNA can carry multiple oncogenes (17). Additionally, ecDNA can also carry immune regulatory genes and inflammation-related genes (3, 13). Gene function enrichment analysis confirmed that genes carried by ecDNA are often upregulated in biological processes such as cell cycle, cell division, and DNA damage, and downregulated in processes related to the immune system (18).

### 2.2 Non-Mendelian segregation mechanisms

**Figure 2:**
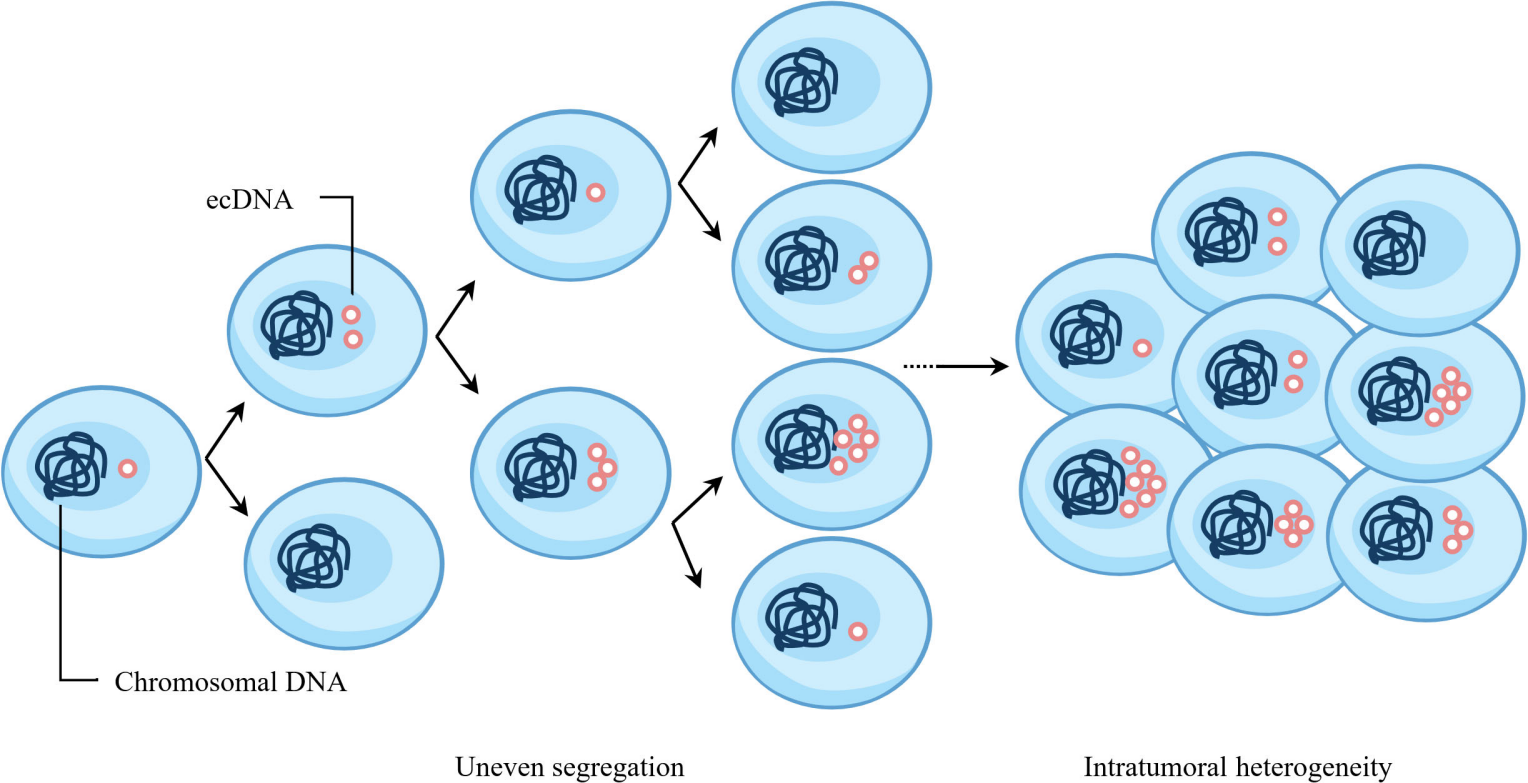
Random segregation of ecDNA promotes intratumoral heterogeneity of cancer. ecDNA does not follow Mendelian inheritance and undergoes random segregation during cell division. After multiple rounds of cell division, the copy number of ecDNA in cells will exhibit significant heterogeneity.

During mitosis, the spindle apparatus pulls the centromeres of chromosomes, guiding their alignment and equal segregation to ensure that daughter cells have identical chromosomal DNA. Multiple studies have shown that the copy number of ecDNA among tumor cells exhibits significant heterogeneity (19, 20). ecDNA does not appear to follow Mendelian inheritance during cell division, differing from chromosomal inheritance patterns (21). FISH-based methods combined with unbiased image analysis have shown that after mitosis in multiple tumor cell lines, the number of ecDNA in daughter cells follows a Gaussian distribution, and the segregation process is independent of tumor type and ecDNA species (22). Subsequently, CRISPR-based ecDNA tagging with live-cell imaging was used to dynamically track ecDNA during the cell cycle, further confirming that ecDNA undergoes random segregation during cell division (22, 23). Analysis of The Cancer Genome Atlas Program (TCGA) database showed that more than 25% of ecDNA-containing (ecDNA+) tumors contain more than two types of ecDNA, and different ecDNA species coexist at copy numbers much higher than expected by chance (14). Therefore, when cells contain multiple ecDNA species, their segregation may not be completely independent and random. Recent studies have shown that when tumor cells contain multiple ecDNA species, cooperative ecDNA species are coordinately inherited through mitotic co-segregation (14). In summary, during cell division, a single type of ecDNA segregates into daughter cells in a binomial random manner, while multiple coexisting ecDNA species can be co-segregated into daughter cells (**Figure 2**).

## 3 Formation of ecDNA

**Figure 3:**
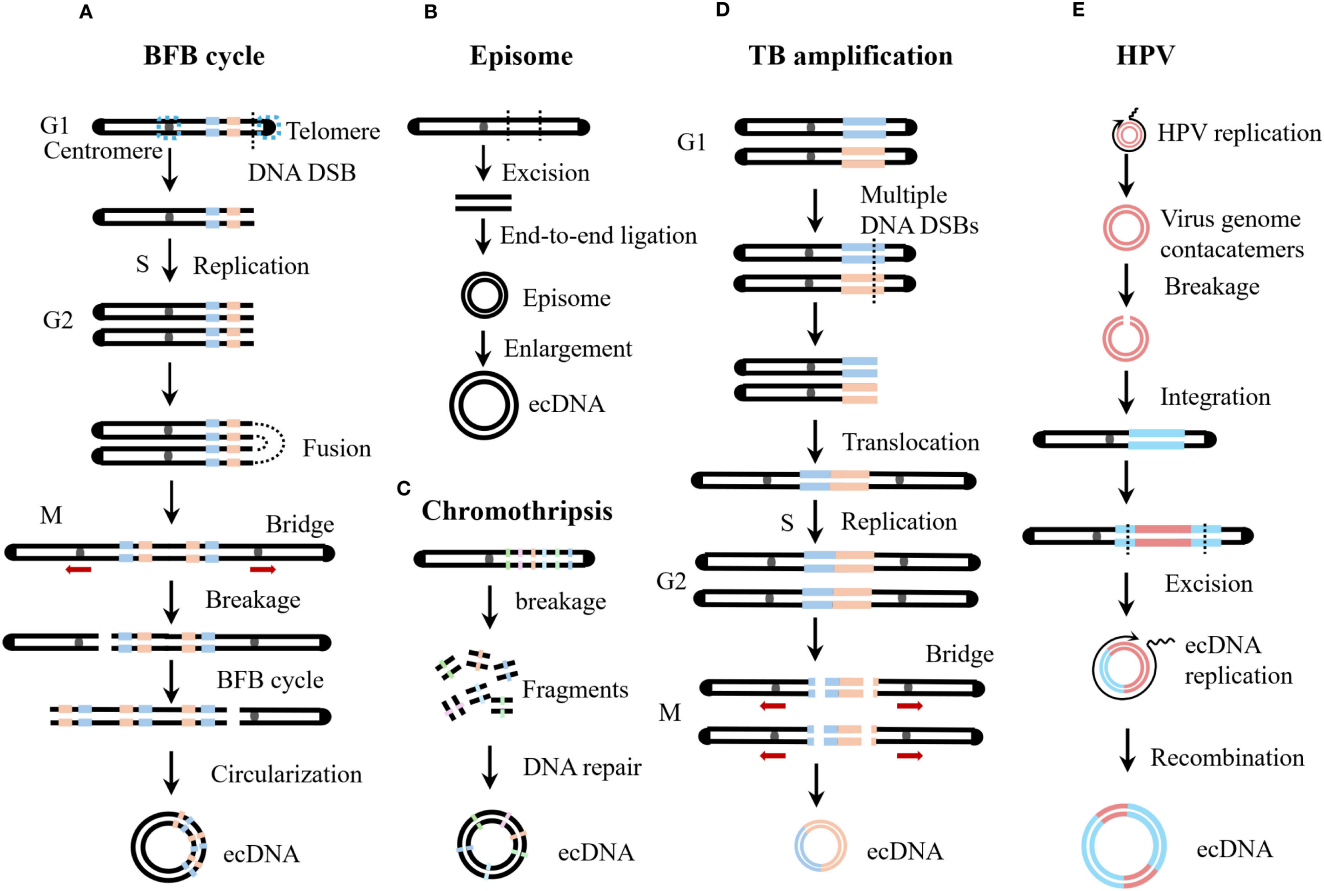
Biogenesis of ecDNA. **(A)** Breakage-Fusion-Bridge (BFB) cycles start during a bridge formation (usually between sister chromatids) as a stabilizing repair intermediate for DNA double-strand break (DSB). Unequal mitotic separation and breakage of the bridged chromosomes creates an inverted duplication on one chromosome, and a deletion on the other. The broken end result in continued BFB cycles until the telomere is re-capped. **(B)** The episome model is also known as the excisional model. Episomes are derived from excisied DNA fragments, and they can enlarge to form ecDNA by over-replication or recombination. The excisional model is divided into the scarring excisional model and the scarless excisional model. **(C)** Chromothripsis generates multiple chromosomal fragments via a single catastrophic event. These fragments may undergo misrepair and illegitimate reassembly, driving either massive chromosomal rearrangements or ecDNA formation. **(D)** Translocation-bridge (TB) amplification occurs in the G1 phase. Inter-chromosomal translocation directly creates the dicentric chromosome. During mitosis, dicentric chromosomes separate to form chromosomal bridges, which break and circularize to form ecDNA. **(E)** Human papillomavirus (HPV) integration drives host genome amplification and structural rearrangement, resulting in virus-host tandem DNA formation. Subsequent excision of viral DNA by host cells generates hybrid virus-host ecDNA.

Since the discovery of ecDNA, researchers have been committed to exploring its origin and formation mechanisms. Analysis of single nucleotide variant (SNV) frequency has shown that ecDNA and chromosomal DNA are haplotypically distinct, providing evidence for the hypothesis that ecDNA originates from chromosomes (24). Currently, several models have been proposed to explain the formation of ecDNA, such as the excisional model, breakage-fusion-bridge (BFB) cycle, translocation-bridge amplification (TB amplification), and chromothripsis. Below, we discuss these models in detail (**Figure 3**).

### 3.1 Excisional model

The excisional model, also known as the episome model, is a simple hypothesis regarding the origin of ecDNA. The excisional model posits that ecDNA originates from double-stranded DNA breaks on chromosomes, so the resulting ecDNA usually have simple structures and low diversity (25). As early as 1988, researchers confirmed that episomes can be directly formed from deleted chromosomal fragments (26). Subsequently, a case of acute myeloid leukemia was reported in which the leukemic cells contained double minutes carrying the *MYC* gene, and a chromosome 8 with a deletion in the *MYC* region was also present, suggesting that the *MYC* in the double minutes originated from chromosome 8 (27). Through next-generation sequencing, single nucleotide polymorphism array, fluorescent *in situ* hybridization, and polymerase chain reaction-based techniques, the genomic structure and evolutionary mechanisms of seven *MYC* ecDNA+ tumor cell lines were analyzed, revealing that ecDNA are gradually formed through multiple steps such as amplification, recombination, and deletion of ancestral episomes from a single chromosome (28). Chromosome conformation capture (Hi-C) analysis showed that spatial proximity is not required for the generation of ecDNA, and long-distance double-stranded breaks can still efficiently form ecDNA (29). In addition, CRISPR-C technology can be used to artificially cut specific genes to construct ecDNA+ cell models (12, 22).

According to whether the chromosome can be accurately repaired after DNA fragment excision, the excisional model is divided into the scarring excisional model and the scarless excisional model. In the scarring excisional model, DNA fragment excision occurs before DNA replication, and the organism connects the chromosomal break ends through nonhomologous end joining (NHEJ) (25). In the scarless excisional model, DNA fragments are usually formed by replication fork breakage, and the missing part of the chromosome is precisely repaired through a homologous recombination-dependent DNA replication process using the normal sister chromatid as a template (25). Deep sequencing of the junctions between ecDNA and chromosomal excision scars revealed that the formation of ecDNA and chromosomal scars is independent, with NHEJ predominantly repairing chromosomal scars, while microhomology-mediated end joining (MMEJ) is more common in ecDNA circularization (29). However, some studies have shown that the breakpoints of circular amplicons often have no sequence homology or only minimal sequence homology (<5 bp), suggesting that NHEJ is the main mechanism for ecDNA formation (30).

### 3.2 Breakage-fusion-bridge cycle

BFB cycles were originally described by Barbara McClintock in 1939 for the fate of a dicentric chromosome during meiotic mitosis and endosperm development in maize (31). BFB events are common in tumors and are a frequent cause of increased oncogene copy number (32, 33). The BFB cycle begins with telomere loss, and chromosomes lacking telomeres or sister chromatids of telomere-deficient chromosomes after replication fuse to form dicentric chromosomes (34). During cell division, dicentric chromosomes are pulled in opposite directions by spindle fibers, forming chromosomal bridges (35). Chromosomal bridges break under mechanical tension during cell division, generating new telomere-deficient chromosomes and broken chromosomal fragments (34). In the absence of telomeres, BFB will continue to occur in subsequent generations of cells until telomeres are restored (32). Studies have shown that there is a strong overlap between oncogenes amplified by BFB cycles and those amplified by ecDNA, suggesting that chromosomal bridge fragments generated by the BFB cycle can circularize to form ecDNA (32).

### 3.3 Translocation-bridge amplification

Chromosomal translocations can also form dicentric chromosomes. During cell division, dicentric chromosomes separate to form chromosomal bridges, which break and circularize to form ecDNA (36). TB amplification elucidates the amplification mechanism of key oncogenes ERBB2 and cyclin D1 (*CCND1*) in breast cancer (36). Chromosomal translocation is the most common cause of chromosomal bridge formation in tumors, and tumors with TB amplification often exhibit loss of heterozygosity (LOH) on the bridge arm (36). In the TB amplification model, dual-LOH occurs on two chromosomal arms, so chromosomal translocation in TB amplification occurs in the G1 phase (36).

### 3.4 Chromothripsis

Chromothripsis refers to the occurrence of a large number of random breaks in one or several chromosomes within a short period (37). Chromosomal shattering does not occur through the accumulation of gene mutations but is a single catastrophic genomic event (38). Fanconi anaemia (FA), a model syndrome of genome instability, is caused by a deficiency in DNA interstrand crosslink repair resulting in chromosome breakage (39). Studies have shown that the FA pathway is a driving factor for chromothripsis, with the core FA complex monoubiquitinating and activating FANCI-FANCD2, which recruits the SLX4-XPF-ERCC1 endonuclease to cleave micronuclear chromosomes, triggering large-scale chromosomal shattering (40). The shattered chromosomal fragments undergo erroneous joining and assembly, leading to extensive chromosomal rearrangement or the formation of ecDNA (41). Chromothripsis is widespread in tumors, with an incidence exceeding 40% in glioblastoma, lung adenocarcinoma, osteosarcoma, and liposarcoma (42). Additionally, approximately 50% of cases with circular amplicons exhibit chromothripsis (30, 43). Some researchers also believe that chromothripsis may arise through TREX1-mediated fragmentation of dicentric chromosomes formed in telomere crisis (44). BFB cycles and chromothripsis are hallmarks of telomere crisis (45). Therefore, chromothripsis and BFB cycles may share a common origin and coexist and promote each other in the formation of ecDNA (30, 43).

## 4 Viral integration and ecDNA biogenesis

HPV is a small, non-enveloped virus with a circular double-stranded DNA genome, and more than 200 genotypes have been identified to date (46, 47). According to its carcinogenicity, HPV is classified into low-risk and high-risk types (48). High-risk HPV infection can lead to malignant tumors such as cervical cancer, vaginal cancer, penile cancer, anal cancer, oropharyngeal cancer, and head and neck cancer (49). After HPV infection, integration of its genome into the host chromatin is a characteristic step in cellular carcinogenesis, ensuring constitutive expression of the E6/E7 oncogenes (50, 51). As mentioned above, HPVOPC contains virus-host hybrid ecDNA (17, 52). Does HPV play a specific role in the formation of virus-host hybrid ecDNA? Studies have shown that HPV integration can mediate amplification and rearrangement of the host genome, altering the local chromosomal structure and forming virus-host tandem DNA (53–55). Subsequently, researchers found that this tandem DNA sequence in various HPV-related tumors exhibits repetitive, diverse, and interrelated structural features, which were named “heterocateny” (56). Heterocateny is driven by the HPV genome, exists both intrachromosomally and extrachromosomally, and its formation process is as follows (1): HPV replication forms unstable viral genome concatemers (2); viral genome concatemers integrate into host DNA (3); host cells excise viral DNA, forming virus-host hybrid ecDNA (4); virus-host ecDNA undergo replication, amplification, and rearrangement, forming diverse ecDNA (5); virus-host ecDNA can recombine into chromosomes again, and undergo further excision, circularization, replication, amplification, and rearrangement, resulting in even more diverse ecDNA (56). In summary, HPV integration into the host genome can drive the formation of virus-host ecDNA, which are variable in size and structurally diverse (57).

## 5 ecDNA promotes oncogene expression

**Figure 4:**
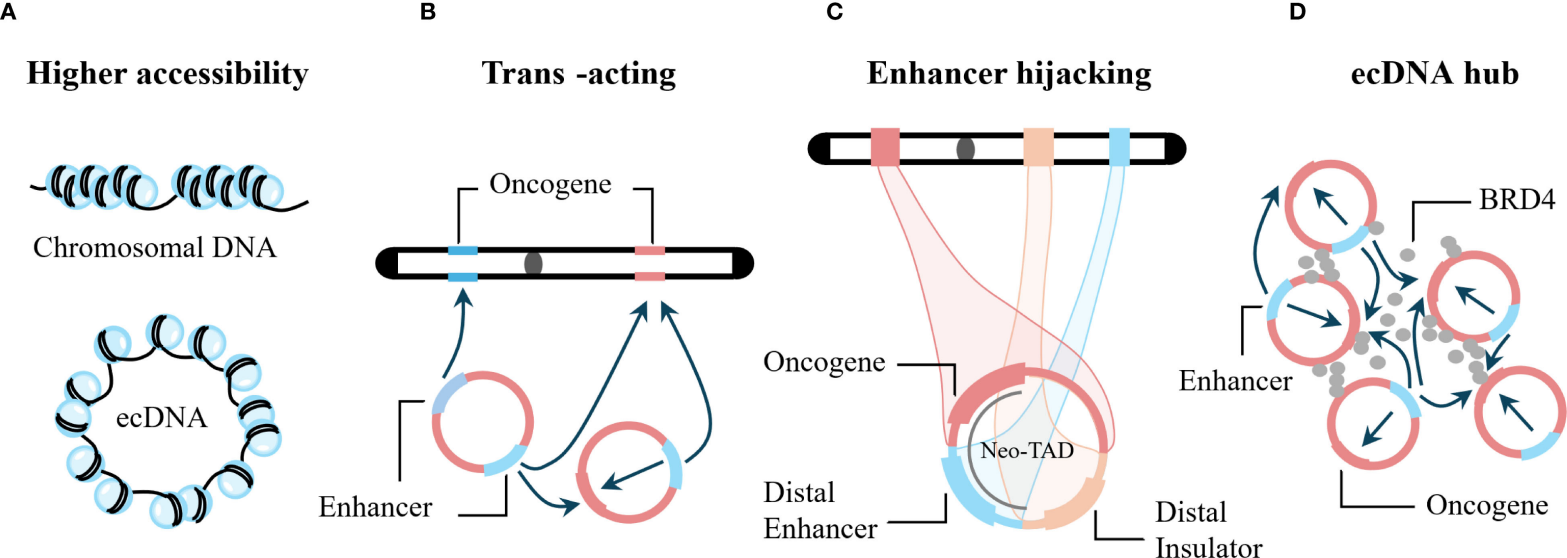
Examples of how ecDNA can promote oncogene expression. **(A)** The nucleosome structure of ecDNA has higher chromatin accessibility, enabling increased transcription. **(B)** ecDNA functions as a trans-acting mobile element that enhances gene expression from both chromosomal loci and other ecDNA molecules. **(C)** ecDNA hijacks both local and distal chromosomal enhancers to elevate oncogene expression. **(D)** ecDNAs assemble into transcriptional hubs that amplify gene transcription across proximal ecDNA molecules through shared regulatory elements, including enhancers.

Pan-cancer analysis shows that oncogenes encoded by ecDNA have the highest expression levels in tumor transcriptomes (15). In fact, the expression levels of *EGFR*, *MYC*, *CDK4*, and *MDM2* genes commonly carried by ecDNA rank in the top 1% of tumor genomes (15). Increased gene expression usually involves increased gene copy number and altered transcriptional regulation. Below, we discuss in detail the mechanisms by which ecDNA promotes increased oncogene expression (**Figure 4**).

### 5.1 Amplification of ecDNA copy number

In tumors, gene amplification is a measure to antagonize anti-cancer treatments by directly increasing the dosage of target proteins (58), or by activating an alternative cell-proliferation pathway (59). More commonly, genes promoting tumorigenesis and progression employ gene amplification to increase their protein level (60). Gene amplification occurs in two forms: linear amplification and circular amplification, manifested as homogeneously staining regions (HSR) and ecDNA, respectively (61). Analysis of 8,068 circularly amplified genes and 6,247 linearly amplified genes in 77 tumor samples showed that the copy number of circularly amplified genes was significantly higher than that of linearly amplified genes (15). Therefore, increased copy number of oncogenes carried by ecDNA is one of the main mechanisms for increased gene expression. In addition, engineered ecDNA can also spontaneously accumulate in primary cells and promote cell proliferation, transformation, immortalization, and drive tumor formation (62).

### 5.2 ecDNA exhibits high transcriptional activity

ecDNA can carry multiple regulatory regions, resulting in a non-linear relationship between transcriptional output and gene copy number. Transcription of ecDNA is very common (63). After normalizing the copy number of oncogenes carried by ecDNA and chromosomal oncogenes, oncogenes on ecDNA still produce more transcripts, significantly higher than those on chromosomes (15, 30). Studies have shown that ecDNA can promote oncogene transcription through non-copy number-dependent mechanisms such as increased chromatin accessibility and enhancer hijacking (64). In addition, as the copy number of ecDNA increases, the transcription level of genes carried by ecDNA also increases accordingly (20). Below, we discuss in detail the mechanisms underlying the high transcriptional activity of ecDNA.

#### 5.2.1 High chromatin accessibility of ecDNA

Chromatin accessibility refers to the physical contact permissibility of nuclear macromolecules with chromatinized DNA, which is mainly determined by the distribution and occupancy of nucleosomes, as well as other DNA-binding factors (65, 66). Accessible chromatin is a hallmark of active DNA regulatory elements (67). The accessible regions comprise only ~2-3% of the whole genome, and more than 90% of these regions are yet to be captured by transcription factors (68). ecDNA is also composed of nucleosome units and possesses chromatin structural features (15). Assay for transposase-accessible chromatin using sequencing (ATAC-seq) and transposase-accessible chromatin with visualization (ATAC-see) experiments have shown that the nucleosome structure of ecDNA is more loosely assembled, lacks higher-order compaction, and has higher chromatin accessibility (15, 64).

#### 5.2.2 ecDNA as mobile enhancers

Gene expression is regulated by genomic enhancers that recruit transcription factors and cofactors to activate transcription from target core-promoters (69). In the HMS001 human papillomavirus-associated oropharyngeal cancer cell line, HPV can integrate into host enhancer regions, forming ecDNA containing enhancer-E6/E7 promoter complexes, and CRISPR interference with this enhancer can reduce E6/E7 expression (52). Therefore, ecDNA can enhance the expression of its own carried oncogenes. Does ecDNA also affect genes at other loci? Studies using artificial enhancer ecDNA to transfect the PC3 prostate cancer cell line (ecDNA−) showed that this ecDNA triggered genome-wide chromosomal gene transcriptional activation in PC3 cells (70). In addition, in cervical cancer cell lines containing HPV virus-host ecDNA with super-enhancers, these super-enhancers can promote strong and extensive intra- and inter-chromosomal interactions (71). The above studies indicate that ecDNA can act as mobile enhancers, facilitating extensive internal interactions and genome-wide chromosomal interactions, thereby broadly promoting gene expression (15, 70).

#### 5.2.3 Enhancer hijacking

Genes can hijack distal enhancers to compensate for the loss of local gene regulatory elements, thereby enhancing gene expression, a phenomenon known as enhancer hijacking (16). Enhancer hijacking can form new topological domains and is an effective mechanism for driving oncogene expression (72, 73). Studies have shown that in the CHP-212 neuroblastoma cell line, *MYCN* on ecDNA can hijack the enhancer element of the tribbles pseudokinase 2 (*TRIB2*) gene (16), *CDX2* on ecDNA in COLO320-DM cells can hijack the *MYC* enhancer (74), and in SNU16 cells, *MYC*, *FGFR2*, *CD44*, and pyruvate dehydrogenase complex component X (*PDHX*) on ecDNA can mutually hijack enhancers (74). In addition, in hematological tumors, ecDNA can also promote oncogene transcription through enhancer hijacking (64). The above studies indicate that enhancer hijacking is common on ecDNA and is an important reason for oncogene overexpression promoted by ecDNA.

#### 5.2.4 Promoter hijacking

ecDNA in COLO320-DM cells contains multiple copies of the long noncoding RNA gene *PVT1*, and *PVT1* often fuses with the *MYC* gene, constituting more than 70% of *MYC* gene transcripts (75). The *PVT1-MYC* fusion gene is formed by the fusion of the *PVT1* promoter and exon 1 with exons 2 and 3 of *MYC*, that is, the *PVT1* promoter and exon 1 replace the *MYC* gene promoter and exon 1 (75). CRISPR interference experiments inhibiting the *PVT1* gene promoter showed that the total amount of *MYC* gene transcripts decreased (75). High-throughput conformation capture with chromatin immunoprecipitation (HiChIP) experiments showed that multiple enhancers can significantly interact with the *PVT1-MYC* promoter, and its H3K27ac signal is higher than that of the classic *MYC* gene promoter (75). In addition, studies of multiple small cell lung cancer (SCLC) cell lines found that the *MYCL* gene on ecDNA can significantly increase MYCL expression by hijacking the *RLF* promoter (76). In summary, ecDNA can increase the expression of related genes by hijacking promoters, facilitating broader contact with enhancers.

#### 5.2.5 ecDNA hub

FISH technology has confirmed that ecDNA in PC3 cells, COLO320-DM cells, SNU16 cells, and HK359 glioma cell lines all exhibit a significant tendency to aggregate in the nucleus (75). Aggregated ecDNA are referred to as ecDNA hubs (75). In addition, *FGFR2* ecDNA and *MYC* ecDNA in SNU6 cells are intertwined in the same hub (75). Bromodomain containing 4 (BRD4) is a member of the bromodomain and extra-terminal domain (BET) family, which also includes BRD1, BRD3, and bromodomain testis associated (BRDT) (77). Live-cell imaging has shown that BRD4 protein is highly enriched in the ecDNA hub of COLO320-DM cells (75). JQ1 is a broad-spectrum BET inhibitor targeting all four BET proteins (77). JQ1 can disperse the ecDNA hub, causing ecDNA to be distributed diffusely, suggesting that BRD4 is a key mediator for the formation and maintenance of the ecDNA hub (75, 78).

Immunofluorescence staining has confirmed that the ecDNA hub in glioblastoma-derived neurosphere cell lines is co-localized with RNA polymerase II (RNAPII), suggesting that the ecDNA hub can promote the aggregation and recruitment of functional transcriptional machinery (23). Transfection of COLO320-DM cells with a *PVT1* promoter-NanoLuc luciferase (PVT1p-nLuc) plasmid confirmed the presence of inter-molecular enhancer-promoter activation in the ecDNA hub and determined that PVT1p can be trans-activated in the ecDNA hub (75). In SNU6 cells, the enhancer on *FGFR2* ecDNA can trans-activate *MYC* gene expression (75). Therefore, co-localization of *FGFR2* ecDNA and *MYC* ecDNA can further promote MYC expression. In COLO320-DM cells, aggregation of *MYC* ecDNA predicts *MYC* pre-mRNA expression levels better than *MYC* copy number (78). In summary, the ecDNA hub increases the spatial proximity between regulatory elements and oncogenes, leading to increased oncogene expression. In addition, after JQ1 disperses the ecDNA hub in COLO320-DM cells, the expression of the *MYC* gene carried by ecDNA also decreases, further confirming that the ecDNA hub can promote oncogene expression (75).

It is worth noting that not all ecDNA in all cells can form ecDNA hubs or exhibit high transcriptional activity. Studies have shown that in glioblastoma stem cells, there is no aggregation or close interaction between ecDNA carrying *EGFR*, *MYC*, and *PDGFR*, nor with transcriptional condensates, and the increase in transcriptional products is due to increased copy number (79). In summary, increased oncogene expression on ecDNA involves multiple mechanisms, which may coexist or exist independently.

## 6 ecDNA promotes tumor heterogeneity, evolution, drug resistance, and poor prognosis

Tumor cells continuously evolve into populations with intratumoral heterogeneity (80). Tumor heterogeneity is caused by genetic, epigenetic, transcriptomic, and phenotypic heterogeneity, and natural selection and Darwinian evolution drive tumor progression and drug resistance on this basis (80, 81). Therefore, tumor heterogeneity is a key factor leading to drug resistance, treatment failure, and death in patients (82). Studies have shown that ecDNA plays an important role in promoting tumor heterogeneity, evolution, and drug resistance (83, 84).

### 6.1 ecDNA copy number, epigenetic, and genetic heterogeneity

As mentioned above, ecDNA does not follow Mendelian inheritance and undergoes random segregation during cell division. Therefore, after multiple rounds of cell division, the copy number of ecDNA in cells will exhibit significant heterogeneity (**Figure 2**) (21, 22). When the cellular microenvironment changes, natural selection enables cells to rapidly accumulate ecDNA carrying oncogenes to cope with adverse environments (22). ecDNA+ tumors adapt faster, exhibit more pronounced intratumoral heterogeneity, and develop drug resistance earlier than BFB-amplified tumors (32). DNA methylation is an important epigenetic modification regulating gene expression (85). In multiple SCLC cell lines, the DNA methylation level in ecDNA+ cells is slightly lower than that in ecDNA− cells (76). Using nanopore sequencing technology, it was found that the methylation level of the EGFR gene promoter region on ecDNA in GBM39 cells is significantly lower than that of the same region on chromosomes (24). Therefore, ecDNA can regulate gene methylation status to achieve higher transcriptional activity.

In glioblastoma, exons 2–7 of *EGFR* are often deleted, resulting in the constitutively active mutant *EGFRvIII* (86). *EGFRvIII* in GBM39 cells is mainly located on ecDNA, while chromosomes usually contain full-length wild-type *EGFR* (24). Therefore, *EGFRvIII* ecDNA in glioblastoma can provide a unique selective advantage for tumor evolution (14). Studies have shown that the expression of apolipoprotein B mRNA editing enzyme catalytic polypeptide 3 (APOBEC3) is significantly higher in ecDNA+ tumors than in ecDNA− tumors, and 31% of samples containing ecDNA exhibit kyklonic events (APOBEC3 kataegis and ecDNA occurring simultaneously) (87). Among all kyklonic events, 41% overlap with known tumor driver genes, resulting in mutations in tumor driver genes (87). Thus, APOBEC3 plays an important role in ecDNA mutation and tumor evolution. In addition, HPV-host ecDNA can undergo multiple rounds of amplification and recombination, forming highly heterogeneous virus-host ecDNA, thereby promoting heterogeneity and clonal evolution in HPV-related tumors (56). The above studies indicate that ecDNA exhibits copy number heterogeneity and species heterogeneity among cells, as well as genetic and epigenetic heterogeneity with chromosomal DNA.

### 6.2 ecDNA promotes selection and drug resistance

Studies have shown that the detection rate of ecDNA is significantly increased in tumor patients receiving chemotherapy and targeted therapy, suggesting that ecDNA may be an adaptive mechanism for tumor cells to cope with treatment pressure (3). *DHFR* gene amplification is the main cause of acquired methotrexate resistance (88). The amplification of the DHFR gene in the methotrexate-resistant HT29 human colon cancer cell line undergoes three stages: pre-amplification, HSR, and ecDNA, with ecDNA being the main driver of resistance (88). After methotrexate treatment of HAP1 cells containing *DHFR* ecDNA, the copy number of ecDNA increased in a strongly dose-dependent manner (22). In urothelial cancer, the *CCND1* gene undergoes amplification through ecDNA-mediated structural variants (SVs), driving cell cycle progression and enhancing cellular adaptability under selective therapeutic pressure (89). Treatment of GBM39-EC (*EGFRvIII* located on ecDNA) and GBM39-HSR (*EGFRvIII* located on HSR) cells with the EGFR tyrosine kinase inhibitor (TKI) erlotinib showed that GBM39-EC cells resist erlotinib by reducing the copy number of *EGFRvIII* ecDNA, while the copy number of *EGFRvIII* in GBM39-HSR cells remains unchanged, and these cells remain sensitive to erlotinib (22). In glioblastoma patients receiving EGFR TKI targeted therapy, tumor cells acquire EGFR TKI resistance by eliminating *EGFRvIII* ecDNA, and *EGFRvIII* ecDNA reappear after drug withdrawal (90). Pancreatic ductal adenocarcinoma (PDAC) is usually difficult to survive in a WNT-deficient environment, and acquired WNT independence can promote PDAC progression (19). In PDAC organoid cultures under WNT-deficient conditions, *MYC* ecDNA+ cells can be selected to adapt to the WNT-deficient environment (19). In addition, GBM39-HSR cells are extremely sensitive to glucose deprivation, while GBM39-EC cells show no significant changes (22). The above studies indicate that ecDNA+ tumors have a stronger selective advantage and can rapidly adapt to changes in the microenvironment when facing metabolic stress or drug treatment, leading to rapid tumor progression and drug resistance.

### 6.3 ecDNA leads to poor prognosis

Studies have shown that patients with ecDNA+ medulloblastoma were more than twice as likely to relapse and three times as likely to die within 5 years of diagnosis (20). After adjusting for tumor type, stage, age, sex, and genomic instability in 14,778 tumor patients (39 types), it was found that ecDNA detection was associated with tumor stage, metastasis, and shorter overall survival (3). Research indicates that reduced expression of MHC class I molecules in ecDNA+ urothelial carcinoma cells enables immune evasion from T cell attack, thereby contributing to poor prognosis in ecDNA+ urothelial cancer patients (91). Moreover, ecDNA can harbor genes regulating immune and inflammatory responses, which is associated with reduced T cell infiltration in cancer patients (3). Therefore, ecDNA may affect tumor progression and prognosis by influencing the expression of immune-related genes, such as inhibiting immune clearance of tumor cells or promoting immune evasion (3, 92). The p53 tumor suppressor protein is a transcription factor that inhibits cell division or survival in response to various stresses (93). *TP53* mutations are associated with enhanced chromosomal instability, including increased amplification of oncogenes and deep deletion of tumor suppressor genes (94, 95). Studies have shown that ecDNA is the main driver of the progression of high-grade dysplasia Barrett’s esophagus (HGD) to esophageal adenocarcinoma (EAC), and *TP53* mutations drive the formation of ecDNA (13). *TP53* mutations are significantly enriched in ecDNA+ endometrial cancer, renal cancer, breast cancer, and medulloblastoma (3, 12, 20). Therefore, some researchers believe that the impact of ecDNA on patient survival is due to *TP53* mutations (20). The overall survival rate of neuroblastoma patients with ecDNA-derived rearrangements is significantly lower than that of patients without ecDNA-derived rearrangements, and the overall survival rate of patients with *MYCN* ecDNA-derived rearrangements is also significantly lower than that of patients without *MYCN* ecDNA-derived rearrangements (96). Therefore, genomic instability caused by the integration of ecDNA into chromosomes may be another mechanism by which it affects patient survival outcomes (97). In addition, in SCLC, ecDNA is the main source of the *RLF-MYCL* oncogenic fusion (76). *RLF-MYCL*, the most common oncogenic fusion in small cell lung cancer, can accelerate transformation and proliferation of murine SCLC and increase metastatic dissemination and the diversity of metastatic sites (98). In summary, multiple studies have confirmed that ecDNA can lead to poor patient prognosis, making it another indicator for prognosis prediction. However, the biological mechanisms by which ecDNA drives tumor progression and metastasis require further study to provide more evidence for the development of future targeted therapies.

## 7 Fate of ecDNA

**Figure 5:**
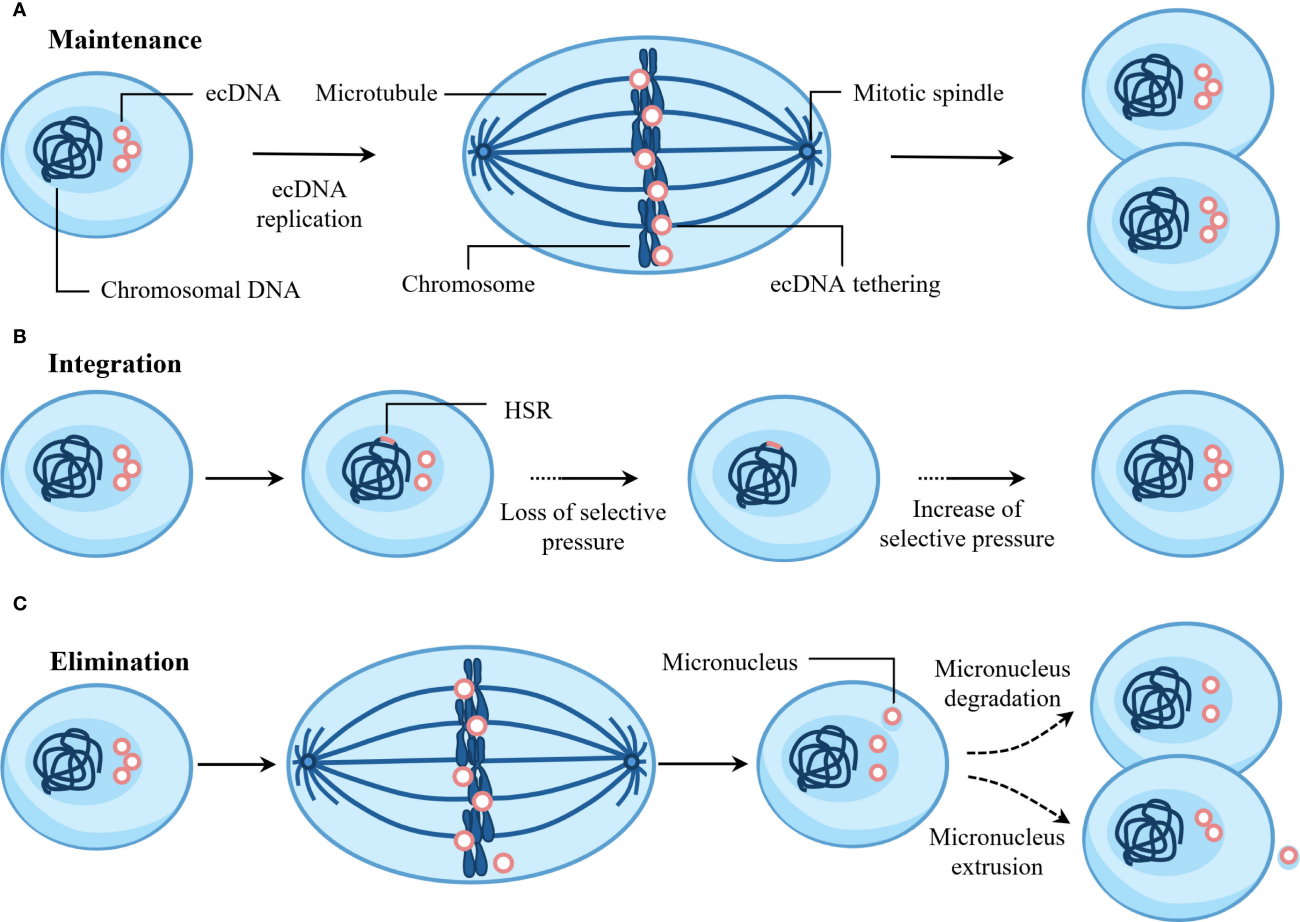
Fate of ecDNA. **(A)** ecDNA is maintained via active replication and mitotic tethering to chromosomes during cell division. **(B)** Random integration of ecDNA into chromosomes generates homogeneously staining regions (HSRs). This integration event is positively selected upon loss of selective pressure. Functioning as ecDNA reservoirs, HSRs can regenerate new ecDNAs when favorable selection pressures reappear. **(C)** ecDNA may undergo elimination through micronucleus formation, followed by extrusion or degradation.

ecDNA plays an important role in tumorigenesis and progression. Can ecDNA persist long-term after abnormal formation in cells? Next, we discuss the fate of ecDNA in cells (**Figure 5**).

### 7.1 Maintenance

ecDNA exhibits autonomous replication and undergoes replication only once during the S phase of the cell cycle (99). ecDNA replication activates the ataxia telangiectasia mutated (ATM)-mediated DNA damage response (DDR) pathway (12). This DDR pathway is essential for ecDNA maintenance, and its inhibition would disrupt ecDNA circularization (12). Furthermore, during mitosis, ecDNA achieves segregation to daughter cell nuclei by attaching to chromosomes – a phenomenon termed “hitchhiking” or “tethering” (100). Research indicates that ecDNA achieves efficient nuclear segregation by tethering to mitotic chromosome ends, and this tethering is essential for ecDNA maintenance (101). In addition, ecDNA can form new, more complex structures through replication, amplification, and rearrangement (41, 102).

### 7.2 Integration

As early as 1985, research revealed that *MYC* amplification in human colonic carcinoma cell lines evolved from ecDNA to HSR on an X chromosome (103). Studies have shown that focal amplification of *BRAF* in the M249 human melanoma cell line resistant to vemurafenib and selumetinib initially exists as ecDNA and can subsequently integrate into chromosomes to form homogeneously staining regions (HSR) (104). In neuroblastoma, most genomic structural rearrangements are caused by the integration of ecDNA into chromosomes (96). For example, after chromosome 2 breaks, ecDNA containing *MYCN*, *NBAS*, and *rs13028343* are formed, and the part of ecDNA containing *NBAS* and *rs13028343* can integrate into chromosome 13, causing the doublecortin like kinase 1 (*DCLK1*) gene to break (96). In addition, JQ1 can induce the integration of ecDNA into chromosomes, leading to the elimination of ecDNA in cells (102).

### 7.3 Elimination

ecDNA usually carries oncogenes, so eliminating ecDNA can induce cell differentiation and reverse the tumor phenotype. Studies have shown that COLO320-DM cells contain ecDNA micronuclei, and low concentrations of hydroxyurea can further induce their formation (105). Hydroxyurea induces DNA double-strand breaks, causing ecDNA to aggregate and lag behind mitotic chromosomes, eventually forming micronuclei (106). Subsequently, CRISPR Cas9 was employed to induce precise double-strand breaks in ecDNA. This approach confirmed that damaged ecDNA is prone to aggregation, and that aggregated ecDNA subsequently detaches from mitotic chromosomes, forming micronuclei (107). In addition, gemcitabine also promotes micronucleus formation of ecDNA in human ovarian cancer cells (108). The MDC1-TOPBP1-CIP2A complex mediates the tethering of chromosomal fragments, allowing them to be transmitted as a whole to daughter cells (109). Therefore, the MDC1-TOPBP1-CIP2A complex may also explain why broken ecDNA are more likely to aggregate, but this requires further research. Moreover, recent studies demonstrate that BRD4 plays a significant role in the nuclear segregation of ecDNA, and inhibition of BRD4 impairs ecDNA clustering during mitotic segregation, ultimately leading to micronucleation (101). Subsequently, ecDNA micronuclei are degraded in cells through autophagy or apoptosis-like processes, or extruded from cells through exocytosis-like mechanisms, becoming the main pathways for ecDNA elimination (105).

## 8 ecDNA as a potential therapeutic target

ecDNA promotes massive transcription of oncogenes and rapid genomic evolution in tumor patients, leading to drug resistance and reduced survival rates. Therefore, ecDNA is an important potential therapeutic target in tumors. Under normal conditions, replication forks on ecDNA exhibit slightly reduced speed and an elevated stalling rate, indicating that they persistently operate under a certain degree of replication stress (110). The reduced fork speed on ecDNA may be associated with increased replication pressure resulting from high-copy gene amplification (111). Studies demonstrate that hydroxyurea can further induce replication stress on ecDNA, exacerbating replication impairment and reducing fork speed, ultimately depleting ecDNA within cells (110). Furthermore, ecDNA undergoes extensive transcription, leading to significantly increased levels of transcription-replication conflicts (63). Such conflicts can cause replication fork reversal and DNA breakage (112). Transcription-replication conflict, replication stress, and DNA damage can drive activation of the S-phase checkpoint (63). The S-phase checkpoint involving checkpoint kinase 1 (CHK1) is essential for fork stability in response to fork stalling (113). The CHK1 protein kinase is essential to ensure genome integrity and cell survival (114). Tang et al. (63) employed a CHK1 inhibitor and CRISPR knockout assays, demonstrating that ecDNA+ tumor cells exhibit heightened sensitivity to CHK1 inhibition, and *CHK1* knockout was shown to induce ecDNA damage and subsequent cell death. Subsequently, the oral CHK1 inhibitor BBI-2779 was further applied in a mouse gastric cancer model containing FGFR2 ecDNA, confirming that BBI-2779 can inhibit gastric cancer growth and cause sustained tumor regression in mice (63). Similar to BBI-2779, BBI-355 is also an oral, potent, selective CHK1 small molecule inhibitor in development as an ecDNA-directed therapy (ecDTx). BBI-355 is also an oral, potent, selective CHK1 small molecule inhibitor in development as an ecDNA directed therapy (ecDTx). BBI-825 is an oral, potent, selective ribonucleotide reductase (RNR) small molecule inhibitor. The combination regimen of BBI-355 and BBI-825 has entered phase 1/2 clinical development for the treatment of patients with proto-oncogene-amplified cancers (NCT05827614). Furthermore, leveraging the unique structural features of ecDNA, we have summarized potential ecDNA-specific therapeutic strategies in **Table 1**.

**Table 1:** Potential targeted therapeutic strategies for ecDNA.

Intervention nodes	Drugs	Description	Refs.
ecDNA biogenesis	PARP inhibitors	DNA ligase 3 PARylation facilitates ecDNA biogenesis. PARP inhibitors block PARylation, and counteract ecDNA-driven drug resistance.	(115, 116)
ecDNA replication	Hydroxyurea	Under replication stress induced by hydroxyurea treatment, ecDNA replication is compromised, leading to altered origin activation, reduced fork velocity and eventual ecDNA depletion from cells.	(110)
	CHK1/CHK2 inhibitors	The CHK protein kinase is essential to ensure ecDNA genome integrity, and CHK inhibitions could trigger preferential cell death in ecDNA+ tumour cells. BBI-355 is currently in clinical trials as a CHK1 inhibitor.	(12, 63)
	ATM/TOP1 inhibitor	ecDNA replication-dependent activation of ATM-mediated DDR and DDR ensures ecDNA maintenance; TOP1 are critical regulators of ecDNA-induced DDR.	(12)
ecDNA clustering	BET inhibitors	BET inhibitors target BRD4 within ecDNA hubs, disrupting hub integrity and thereby reducing intermolecular interactions among ecDNA molecules, ultimately suppressing ecDNA-driven gene expression.	(75)
ecDNA micronucleation	Hydroxyurea	Low-dose hydroxyurea induces ecDNA clustering by promoting DDR. These clustered ecDNAs subsequently detach from anaphase chromosomes, ultimately promoting chromosomal micronucleation.	(106)
	Gemcitabine	Gemcitabine is able to decrease the number of ecDNA in cells at a 7500X lower concentration than the commonly used cancer drug hydroxyurea.	(108)

PARP, poly ADP-ribose polymerase; CHK, checkpoint kinase; ATM, ataxia telangiectasia mutated; TOP1, Topoisomerases; BET, bromodomain and extra-terminal domain; DDR, DNA damage response; BRD4, biromodomain containing 4.

## 9 Conclusions and perspectives

Currently, the detection of ecDNA primarily relies on two complementary approaches: DNA sequencing and imaging-based technologies. High-throughput sequencing enables comprehensive characterization of ecDNA sequence composition and dynamic alterations, yet it remains limited in resolving spatial organization and intercellular variability. In contrast, advanced imaging techniques allow direct visualization and real-time tracking of ecDNA dynamics but lack the capacity to provide precise sequence-level information. Furthermore, ecDNA detection rates vary substantially across cancer types, and the vast diversity of ecDNA-associated oncogenes introduces significant complexity for the development of ecDNA-targeted therapeutics. Adding to these challenges, the profound intratumoral heterogeneity driven by ecDNA through non-Mendelian inheritance mechanisms substantially reduces the diagnostic reliability of single-time tissue biopsies or liquid biopsies.

Future advances are likely to emerge from integrative strategies that combine high-resolution imaging modalities—such as three-dimensional (3D) reconstruction and live-cell imaging—with next-generation and single-molecule sequencing platforms. Such multimodal approaches are expected to provide a systematic understanding of the spatiotemporal dynamics of ecDNA and its influence on tumor evolution, clonal selection, and treatment response. In parallel, therapeutic strategies may increasingly focus on key regulatory nodes that govern ecDNA biogenesis and maintenance, offering potential broad-spectrum targets across multiple tumor types. Additionally, multi-region sampling and single-cell sequencing are anticipated to mitigate false-negative results associated with intratumoral heterogeneity, thereby improving the sensitivity and reliability of ecDNA detection.

In summary, although ecDNA was first described over six decades ago, its critical roles in oncogenesis and cancer progression have only recently gained widespread recognition. A growing body of evidence demonstrates that ecDNA serves as a major driver of oncogene amplification, genomic instability, intratumoral heterogeneity, and therapeutic resistance. Ongoing studies are progressively elucidating the biological processes underlying ecDNA formation, maintenance, clustering, and clearance, laying a theoretical foundation for the development of ecDNA-targeted interventions, with several candidate compounds currently advancing into clinical trials. Nonetheless, key questions remain unresolved regarding the cellular origins, transcriptional regulation, and three-dimensional spatial architecture of ecDNA, highlighting the urgent need for further mechanistic investigations and technological innovations.
